# Woman’s Place in Medicine*Remarks of Dr. Maud J. Frye at the banquet of the Alumni Association of Buffalo University Medical College, April 27, 1897, to the sentiment “Me judice.”

**Published:** 1897-08

**Authors:** 


### WOMAN'S PLACE IN MEDICINE.*

I wish to present to you to-night some thoughts concerning woman's place in medicine; the limit of her work, the line which she will oftenest follow.

Whether we like to confess it or not, woman's physical organization necessarily sets a limit to her achievements. The women of Colorado who propose to serve in the National Guard of that progressive State, will probably remain to the end as they have been from the beginning, a home guard. In reckoning the chances of woman's success when she comes into competition with man, the physical factor has always to be taken into account. The medical work which makes the least demand on woman's physique is, other things being equal, that in which she will best succeed.

It is, however, concerning a feature of women's mental equipment for work in medicine that I wish especially to speak. I do not expect many of you to agree with me, yet I ask you to think on these things. We must acknowledge between the average man and the average woman an unlikeness of mental capacity comparable to their physical unlikeness, a sex in mind, if I may use the term. This is not equivalent to saying that man is superior to woman. There is equality with diversity. The elm is not better than the oak, nor the oak than the elm, but each is good of its kind. There are mental processes which are identical in the sexes, there are others which may oftentimes give advantage to the woman in the work of the physician. Does the women's way of seeing and doing ever handicap her? Let us try and answer the question.

There is one quality which more than any other makes for success in certain lines of medical work. It is the type of courage more or less common to all men, rarely found in women—the courage which dares. It is the courage of the soldier and the explorer. It is the courage which makes possible the great surgeon.

On the other hand, through all time woman's genius has given this character to her work, that it has not turned to great deeds, but has contented itself with the humbler and often holier, tasks of the home, the school-room, and the sick chamber. Her courage makes possible trained nursing, and in the Christian world is the right arm of the church. It is the courage whose other name is patient endurance.

In the early days when the woman who began the study of medicine was of the heroic type, her success was assured from the beginning, for the courage which dared to commence the struggle was equal to any hardship or any emergency which

*Remarks of Dr. Maud J. Frye at the banquet of the Alumni Association of Buffalo University Medical College, April 27, 1897, to the sentiment "Me judice."

arose later. She has opened the way for the woman of average ability, the woman who but for her pioneer work would be teacher or nurse. The average woman has not, as a rule, the daring of an Elizabeth Blackwell. The test of fitness for medical work which college and early professional life gave the one is denied the other, and the open door to medicine is not altogether gain.

The lack of daring does not make a physician less trustworthy, nay rather she may be more worthy of trust, but the lack does not work to her material advantage. There are departments of medicine, notably bacteriology, for which not only woman's type of courage, but her whole manner of training, from her youth up, should eminently fit her, and in such work as this I believe she will excel. But the woman who gains wealth and fame in the practice of general medicine or surgery will be the woman who possesses, without necessarily losing any of her outward womanliness, the masculine type of courage. She is the woman who would rather be wrong than not try. The average woman would rather not try than risk being wrong.

There is a special message which I wish to give to the young women receiving their diplomas to-night. It is concerning your work among women. Whether you intend to be gynecologist or not, every woman who enters your office expects you to be. Setting aside your rights to do whatever you can and desire to do, the fact that women wish women physicians is the only reason for you being such. You will come to feel later the great power for good—not altogether in a professional way, but as a friend and counselor—which is yours, for the good woman who is a good physician has before her, in teaching to her sex right ways of living, and true views of life, a work which will bring satisfaction to herself, and blessing to others.

To the end of time there is to be marrying and giving in marriage, and, except for the woman of unusual talent, wifehood and motherhood are of themselves a vocation. Because of this, and because other occupations, demanding less of her physically and mentally, and offering quicker success, are open to women, the number of women physicians will always be few. Yet whether we ever occupy a great place in medicine or not, a place is ours. We are better women for the knowledge of humanity which our work has given us. May we not hope that mankind will be the better, too, for our having wrought!

"All service is the same with God—
To God, whose puppets, best and worst,
We are; there is no last nor first."

